# ProbBreed: a novel tool for calculating the risk of cultivar recommendation in multienvironment trials

**DOI:** 10.1093/g3journal/jkae013

**Published:** 2024-01-18

**Authors:** Saulo F S Chaves, Matheus D Krause, Luiz A S Dias, Antonio A F Garcia, Kaio O G Dias

**Affiliations:** Department of Agronomy, Federal University of Viçosa, Viçosa 36570-900, Brazil; Department of Agronomy, Iowa State University, Ames, IA 50011, USA; Department of Agronomy, Federal University of Viçosa, Viçosa 36570-900, Brazil; Department of Genetics, Luiz de Queiroz College of Agriculture, University of São Paulo, Piracicaba, 13418-900, Brazil; Department of General Biology, Federal University of Viçosa, Viçosa 36570-000, Brazil

**Keywords:** risk, Bayesian model, genotype-by-enviroment interactions, cultivar recommendation

## Abstract

Neglecting genotype-by-environment interactions in multienvironment trials (MET) increases the risk of flawed cultivar recommendations for growers. Recent advancements in probability theory coupled with cutting-edge software offer a more streamlined decision-making process for selecting suitable candidates across diverse environments. Here, we present the user-friendly ProbBreed package in R, which allows breeders to calculate the probability of a given genotype outperforming competitors under a Bayesian framework. This article outlines the package’s basic workflow and highlights its key features, ranging from MET model fitting to estimating the per se and pairwise probabilities of superior performance and stability for selection candidates. Remarkably, only the selection intensity is required to compute these probabilities. By democratizing this complex yet efficient methodology, ProbBreed aims to enhance decision-making and ultimately contribute to more accurate cultivar recommendations in breeding programs.

## Introduction

Plant breeding programs routinely evaluate experimental genotypes in multienvironmental trials (MET). The phenotypic manifestation in MET for quantitative traits is shaped by genotype-by-environment interactions (GEI), which complicates selection due to crossover (complex) interactions (Cooper and DeLacy 1994; Lynch and Walsh 1998). Neglecting GEI increases the risk of selecting a genotype that performs poorly in specific environments or mega-environments (regions). Thus, exploring GEI is critical for cultivar recommendation in the target population of environments (TPE).

Several studies used the frequentist framework to compute a measure of risk to rank genotypes in MET (Barah *et al*. 1981; Mead *et al*. 1986; Eskridge *et al*. 1991; Annicchiarico 1992). More recently, Dias *et al*. (2022) proposed a novel Bayesian method that employs the posterior distribution to get Hamiltonian Monte Carlo estimates of performance and stability probabilities. Their core ideas are to assess the predictability of an experimental genotype’s performance through its probability of being amongst the selected genotypes in a global (marginal, across environments) or specific context (conditional, within environments or mega-environments); and the probability of a selection candidate having an invariant performance across environments. The method also provides pairwise probabilities, useful for direct comparison of experimental genotypes, or experimental genotypes versus check cultivars.

The package ProbBreed was built upon the method of Dias *et al*. (2022) to allow the application of probability theory to cultivar recommendation in MET. Its underlying method is intuitive for plant breeders for 2 main reasons. First, it emulates a situation usually faced by growers: choosing cultivar(s) that are likely to perform well for the next cropping season. Second, probabilities (marginal or conditional) are calculated according to the intensity of selection, which is also part of plant breeders’ routine. Furthermore, and in contrast with biplot-based methods (Yan *et al*. 2000), our method is straightforward given the sole metric used for selection is the calculated probabilities.

Plant breeding programs can benefit from using our probabilistic approach to perform a more rapid and effective decision-making process toward cultivar recommendation for a TPE. Thus, we present the open-source R (R Core Team 2023) package ProbBreed, a user-friendly tool that democratizes the method from Dias *et al*. (2022), regardless of the user’s programming abilities. We first provide an overview of the Theory behind ProbBreed and describe the Motivating example contained within the package. Finally, in *Results and discussion*, we illustrate a workflow of the package’s usage, employing the described dataset.

## Methods

### Theory

When analyzing data from MET, the main goal is to select high-performance genotypes with stable phenotypic responses across environments, given a selection intensity. Naturally, selecting and recommending experimental genotypes to a TPE encompasses latent risks that plant breeders assume. The probabilities proposed by Dias *et al*. (2022) allow considering these risks when performing the selection. We detail these probabilities below.

#### Probability of superior performance

Consider a dataset in which *J* genotypes (j=1,2,…,J) were evaluated at *K* environments (k=1,2,…,K) with *y* observed phenotypes. Let *Ω* be a subset of the high-performance selected genotypes according to the intensity of selection. A given genotype *j* will belong to *Ω* if its genotypic marginal value (${\hat{g}}_{j}$) is high (or low) enough compared to its peers. We can emulate the occurrence of *S* trials (s=1,2,…,S) with Bayesian models by leveraging Monte Carlo discretized samples from the posterior distributions of the fitted Bayesian models. Then, the probability of the jth genotype belonging to *Ω* is its ratio of success (${\hat{g}}_{j}\in \Omega$) events over the total number of sampled events [$S=({\hat{g}}_{j}\in \Omega )+({\hat{g}}_{j}\notin \Omega )]$, defined as follows:


(1) $$\text{Pr}({\hat{g}}_{j}\in \Omega |y)=\frac{1}{S}\sum_{s=1}^{S}I({\hat{g}}_{j}^{\left(s\right)}\in \Omega |y)$$

where $I({\hat{g}}_{j}^{\left(s\right)}\in \Omega |y)$ is an indicator variable that can assume 2 values: (1) if ${\hat{g}}_{j}\in \Omega$ in the sth sample and (0) otherwise.

Similarly, the conditional probability of superior performance can be applied to individual environments. Let $\Omega_{k}$ represent the subset of superior genotypes in the kth environment, so that the probability of the $j\text{th}\in \Omega_{k}$ can be calculated as follows:


(2) $$\text{Pr}({\hat{g}}_{\;jk}\in \Omega_{k}|y)=\frac{1}{S}\sum_{s=1}^{S}I({\hat{g}}_{\;jk}^{\left(s\right)}\in \Omega_{k}|y)$$

where $I({\hat{g}}_{\;jk}^{\left(s\right)}\in \Omega_{k}|y)$ is an indicator variable mapping success (1) if ${\hat{g}}_{\;jk}^{\left(s\right)}$ exists in $\Omega_{k}$, failure (0) otherwise, and ${\hat{g}}_{\;jk}^{\left(s\right)}={\hat{g}}_{j}^{\left(s\right)}+{\hat{ge}}_{\;jk}^{\left(s\right)}$. Note that when computing conditional probabilities (i.e. conditional to the kth environment or mega-environment), the interaction of the jth genotype with the kth environment is accounted.

The pairwise probabilities of superior performance can also be calculated across or within environments. This metric assesses the probability of the jth genotype being superior to another experimental genotype or a commercial check. The calculations are as follows:


(3) $$\text{Pr}({\hat{g}}_{\;j}>{\hat{g}}_{\;j^{\prime }}|y)=\frac{1}{S}\sum_{s=1}^{S}I({\hat{g}}_{\;j}^{\left(s\right)}>{\hat{g}}_{\;j^{\prime }}^{\left(s\right)}|y)$$

(4) $$\text{Pr}({\hat{g}}_{\;jk}>{\hat{g}}_{\;j^{\prime }k}|y)=\frac{1}{S}\sum_{s=1}^{S}I({\hat{g}}_{\;jk}^{\left(s\right)}>{\hat{g}}_{\;j^{\prime }k}^{\left(s\right)}|y)$$

Note that equations 3 and 4 are set for when the selection direction is positive (i.e. the aim is to increase the trait value). If the selection is negative, > can simply be switched by <. Equation 3 computes the pairwise probabilities across environments, while equation 4 within environments.

#### Probability of superior stability

Probabilities of superior performance highlight high-performance genotypes. For stability, the probability of superior stability is more adequate. This metric can be directly compared to the method of Shukla (1972): a stable genotype is the one that has a low variance of the GEI effects [$\text{var}(\hat{ge})$]. Using the same probability principles previously described, the probability of superior stability is given as follows:


(5) $$\text{Pr}[\text{var}({\hat{ge}}_{\;jk})\in \Omega |y]=\frac{1}{S}\sum_{s=1}^{S}I[\text{var}({\hat{ge}}_{\;jk}^{\left(s\right)})\in \Omega |y]$$

where $I[\text{var}({\hat{ge}}_{\;jk}^{\left(s\right)})\in \Omega |y]$ indicates if $\text{var}({\hat{ge}}_{\;jk}^{\left(s\right)})$ exists in *Ω* (1) or not (0). Note that this probability can only be computed across environments since it depends on $\text{var}({\hat{ge}}_{\;jk})$. Pairwise probabilities of superior stability are also computed in the context of stability:


(6) $$\text{Pr}[\text{var}({\hat{ge}}_{\;jk})<\text{var}({\hat{ge}}_{\;j^{\prime }k})|y]=\frac{1}{S}\sum_{s=1}^{S}I[\text{var}({\hat{ge}}_{\;jk}{)}^{\left(s\right)}<\text{var}({\hat{ge}}_{j^{\prime }k}{)}^{\left(s\right)}|y]$$

#### Joint probability of superior performance and stability

The joint probability of the occurrence of independent events is the product of the individual probabilities. The estimated genotypic main effects and the variances of GEI effects are independent due to the design of linear models, thus the joint probability of superior performance and stability are given as follows:


(7) $$\text{Pr}[{\hat{g}}_{j}\in \Omega \cap \text{var}({\hat{ge}}_{\;jk})\in \Omega ]=\text{Pr}({\hat{g}}_{j}\in \Omega )\times \text{Pr}[\text{var}({\hat{ge}}_{\;jk})\in \Omega ]$$

The estimation of probabilities in this section is closely related to some key questions that are part of plant breeding programs’ daily routine, such as “what is the risk of recommending a selection candidate for a TPE?”, or “how probable is it that a given selection candidate perform similarly across environments?”, or even “what is the probability of a selection candidate having better performance (or more stable performance) than a check cultivar in the TPE and in specific environments?”.

### Motivating example

We demonstrate the application of ProbBreed using a dataset (named soy) from the USDA Northern Region Uniform Soybean Tests, which is a subset of the data used by Krause *et al*. (2023). It contains the empirical best linear unbiased estimates (column named “Y” in the data frame) of genotypic means of the seed yield from 39 experimental genotypes (“G01” to “G39” in the column named “Gen” in the data frame) evaluated in 14 locations (“E1” to “E14” in the column named “Loc” in the data frame) across 3 mega-environments (“R1”, “R2”, and “R3” in the columns named “Reg” in the data frame). The analysis was performed on a computer with 8 GB of RAM and a 12th Gen Intel Core i7-1255U processor, with a base frequency of 1.70 GHz. Computational time was recorded with the get_elapsed_time function from the rstan package.

## Results and discussion

### Bayesian MET models

The first step is to fit the Bayesian MET model using the bayes_met function. Internally, the Bayesian models are fitted using rstan, a package that links Stan to R (Stan Development Team 2023a, 2023b). Stan is a probabilistic library set in C++ language that uses the No-U-turn Sampler (Hoffman and Gelman 2014) to automatically tune up the Hamiltonian Monte Carlo algorithm by eliminating the need to specify the number of leapfrog updates. This avoids the random-walk behavior and improves computational efficiency. For more details about the No-U-turn Sampler and its advantage over regular Hamiltonian Monte Carlo algorithm, see Hoffman and Gelman (2014) and Nishio and Arakawa (2019).

**Fig. 1.**
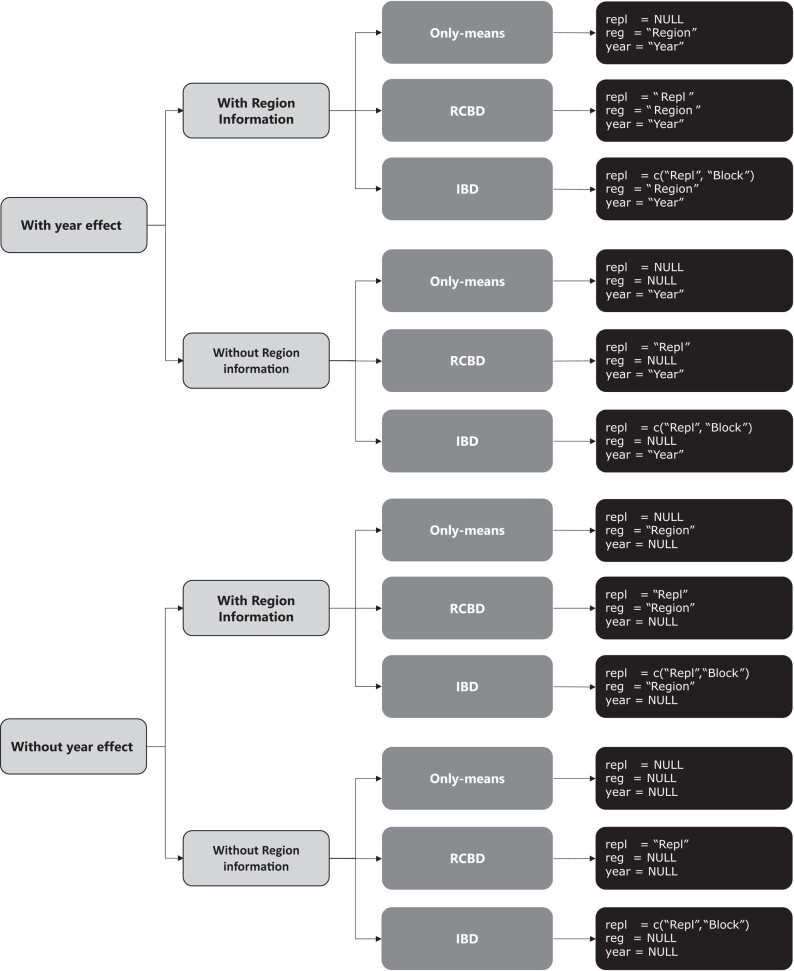
Options to declare replications and/or blocks (repl), years (year), and regions (reg) effects in the bayes_met function. Users must substitute Repl, Block, Year, and Region with the name of the column that contains the information about replicates, block nested in replicates (if applicable), year (if available), and region (if available). RCDB, randomized complete block design; IBD, incomplete block design.

Currently, there are twelve models implemented in ProbBreed. These models differ according to the considered information regarding locations, years and breeding regions (Fig. 1). Additionally, one might consider the collective information from a combination of environmental factors, such as the location–year combination for instance, as constituting an “environment”. Models that consider the information of years are a novelty in relation to Dias *et al*. (2022), see Appendix A for further information. These models also differ regarding the experimental design: entry-mean (i.e. adjusted means), randomized complete block design (RCBD), and incomplete block design (IBD). For example, the soy dataset has information on breeding regions (or mega-environments) and the reported phenotypes are empirical best linear unbiased estimates of genotypic means (i.e. entry-mean basis). The function bayes_met is detailed in Box 1:

Box 1. **Usage of function bayes_met.**

mod = bayes_met(data = soy,

gen = "Gen",

loc = "Loc",

repl = NULL,

reg = "Reg",

year = NULL,

res.het = FALSE,

trait = "Y",

iter = 40000,

cores = 4,

chains = 4)

In summary, users may choose which model to use based on their dataset and by changing the arguments year, reg, and repl (Fig. 1). Users might consider an “environment” as a composite of multiple environmental factors rather than differentiating individual components, as demonstrated by (loc = “Loc” and Reg = “Reg”). In this case, only the loc argument would be employed, such as loc = “Environment”, while year = NULL and reg = NULL. Note from Box 1 that bayes_met has an additional argument that controls if residual variances should be considered homogeneous (res.het = TRUE) or heterogeneous (res.het = FALSE) across locations (or environments). It is noteworthy that even when breeding regions are accounted for in the model and res.het = TRUE, the residual variances are still considered heterogeneous only across locations. Users may also control the number of iterations (iter) and Markov chains (chains). The argument cores determines whether Markov chains run in parallel (cores>1) or not (cores = 1). Each Markov chain runs the specified number of iterations set by the user independently, and, by default, half of them are reserved for the burn-in process. The function supports additional arguments passed to the sampling function of rstan. This allows advanced users to modify parameters such as the number of burn-in iterations, the frequency of saving samples, and other default settings that influence the behavior of the sampler. Users can also define initial values, specify parameters of interest, and select the preferred sampling algorithm. Changing these parameters can aid in fixing convergence and mixing issues (see *On warnings about mixing and convergence issues*). bayes_met documentation has more details on these arguments.

The assumptions of the models implemented in bayes_met have some presets described in detail by Dias *et al*. (2022). In summary, y∼N(E[y],σ), where E[y] depends on the models’ choice. The prior probability distributions of the model effects are $x∼N(0,S^{\left[x\right]})$, where *x* can be any effect but the error, with hyperprior $S^{\left[x/\sigma \right]}∼\text{HalfCauchy}(0,\phi )$. *N* and HalfCauchy represent the Normal and Half-Cauchy distributions, respectively, where the former is constrained to be positive (Gelman *et al*. 2013). The global hyperparameter *ϕ* is defined as ϕ=max(y)×10. The error term has the sampling variance $\sigma ∼\text{HalfCauchy}(0,S^{\left[\sigma \right]})$ for homogeneous residual variances, and $\sigma_{k}∼\text{HalfCauchy}(0,S^{\left[\sigma_{k}\right]})$ for heterogeneous residual variances. The weakly informative prior distributions with their respective hyperpriors allow the model to take full advantage of the data to infer the posterior distribution.

#### On warnings about mixing and convergence issues

By default, rstan detects and warns users of any potential mixing and convergence issues on the fitted model. Usual problems are—but are not limited to—divergent transitions after warm-up, large potential scale reduction factor $\left(\hat{R}\right)$, and insufficient bulk and tail effective sample size. A detailed tutorial on these problems and how to deal with them is available in https://mc-stan.org/misc/warnings.html. We recommend other tools to explore the model’s output and easily detect and solve complications, namely the packages posterior (Vehtari *et al*. 2021; Bürkner *et al*. 2023), bayesplot (Gabry *et al*. 2019) and shinystan (Gabry and Veen 2022). It is worth mentioning that even though rstan is conservative in identifying abnormalities in model fitting, models with alleged imperfect mixing and convergence can still yield acceptable results. We recommend examining the goodness-of-fit diagnostics of the extr_outs (as described in the next section) before making any adjustments to the model or default parameters. If bayes_met shows warnings, but the diagnostics of extr_outs does not indicate grave issues, one may carry on with the analysis.

### Posterior effects and goodness-of-fit diagnostics

After fitting a Bayesian model, the information from the posterior distribution is accessed with the extr_outs function as follows (Box 2):

Box 2. **Usage of function extr_outs.**

outs = extr_outs(data = soy,

trait = "Y",

model = mod,

probs = c(0.05, 0.95),

check.stan.diag = TRUE,

verbose = TRUE)

**Fig. 2.**
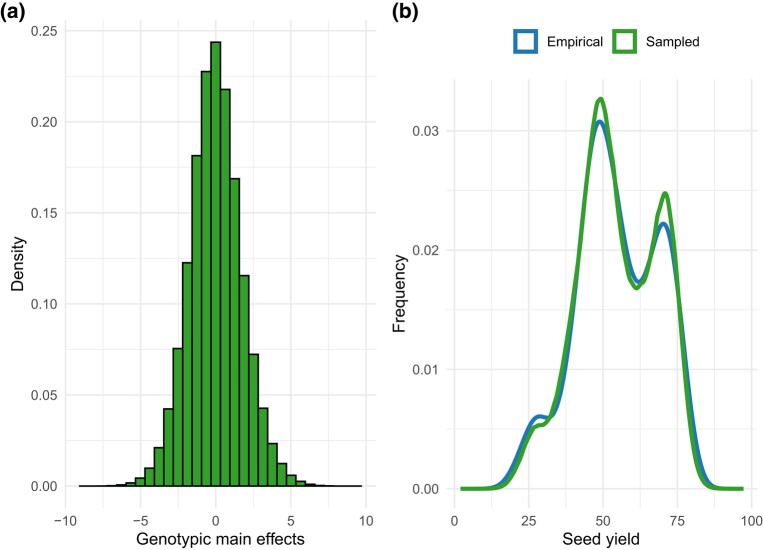
Histogram of the posterior genotypic main effects a) and density plot of the data generated in comparison to the distribution of the real data b). All plots were built with ggplot2 (Wickham 2016).

This function extracts the posterior distributions, the maximum values *a posteriori*, and the data generated by the model. probs is a vector with 2 probabilities in the decimal scale used to calculate the highest posterior density (HPD) interval of the variance components (Table 1). mod is the model fitted using bayes_met. extr_outs uses the posterior distributions and the data generated by the model to build plots that allow an overview of the model’s goodness-of-fit (Fig. 2). The function builds histograms (Fig. 2a) and density plots (Fig. 2b), which provide a visualization of the posterior effects’ distribution; and trace plots, useful for detecting problems with the convergence of each chain. Figure 2b is particularly handy to assess if the model was able to generate data with a similar distribution to the real data. extr_outs provides further diagnostic plots when the argument check.stan.diag is set to TRUE. Internally, these plots are built using the stan_diag function. Further options are available in stan_diaghelp page.

**Table 1.** Estimates of variance components of the declared effects, and their respective standard deviation (SD), naive standard error (Naive SE), and inferior and superior high posterior density interval [HPD (0.05) and HPD (0.95), respectively].

Components	Variance	SD	Naive SE	HPD (0.05)	HPD (0.95)
Genotype (G)	3.314	1.392	0.005	1.39	5.822
Location (L)	251.972	138.611	0.49	107.984	502.227
G×L	6.861	5.47	0.019	0.187	16.328
Region (R)	3181.473	42228.65	149.301	0.772	8138.598
G×R	1.139	1.036	0.004	0.014	3.145
Residual	11.179	5.447	0.019	2.045	18.558

In addition to the referred plots, goodness-of-fit parameters such as the Bayesian “*P*-values” of test statistics, the Watanabe–Akaike information criterion (WAIC2) (Watanabe 2013), and the mean ($\hat{\text{R}}$) (Gelman and Rubin 1992) are provided by extr_outs (Table 2). The WAIC2 has a similar interpretation as AIC (the lower, the better), and it is useful to compare different models. The $\hat{\text{R}}$ evaluates the equilibrium among chains, i.e. if all chains converged to a common distribution. In fact, it is the ratio between the average variance of samples within chains to the variance across chains, so values closer to one indicate that these variances are similar, which is desirable (Fabreti and Höhna 2022). The Bayesian “*P*-values” are computed as the probability of a given test statistic (the mean, for example) being higher in the generated data than in the real data. If the generated data resembles the observed data, Bayesian *P*-values are expected to be far from the extremes (0.99 or 0.01) (Gelman *et al*. 2013). A Bayesian *P*-value closer to 0.5 is desirable (Dias *et al*. 2022). When check.stan.diag = TRUE, extr_outs provides specific diagnostics on possible divergent transitions, tree depth problems and the Bayesian fraction of missing information (BFMI) values of each chain.

**Table 2.** Goodness-of-fit parameters: Bayesian “*P*-values” of test statistics [maximum, minimum, median, mean, and standard deviation], the effective number of parameters, WAIC2, potential scale reduction factor ($\hat{R}$), and effective sample size.

Parameter	Value
*P*-value of the maximum	0.9689
*P*-value of the minimum	0.2614
*P*-value of the median	0.6710
*P*-value of the mean	0.5029
*P*-value of the std. deviation	0.5256
Effective number of parameters	134.068
WAIC2	2550.87
$\hat{\text{R}}$	1.0192
Effective sample size	0.05

### Probabilities

The pipeline finishes with the prob_sup function, which computes probabilities of superior performance and superior stability of the selection candidates. For the soy dataset, the following command line was used (Box 3):

Box 3. **Usage of function prob_sup.**

results = prob_sup(data = soy,

trait = "Y",

gen = "Gen",

loc = "Loc",

mod.output = outs,

reg = "Reg",

year = NULL,

int = .2,

increase = TRUE,

save.df = FALSE,

interactive = FALSE,

verbose = TRUE)

**Fig. 3.**
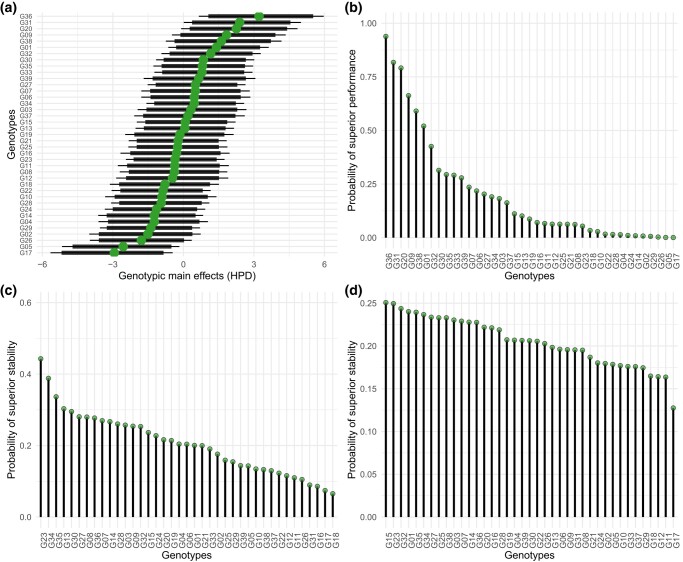
HPD of the posterior genotypic main effects a), probability of superior performance across environments b), and probability of superior stability across locations c), and regions d). The dots at a) are the maximum posterior, and the thick and thin lines at a) represent the 95 and 97.5% HPD intervals, respectively. The *x*-axis of b), c), and d) are sorted in decreasing order considering the computed probabilities. All plots were built with ggplot2 (Wickham 2016).

In this example, we applied a 20% selection intensity (int = .2) and our goal was to increase the average seed yield (increase = TRUE) in the selected panel. These 2 pieces of information dictate how probabilities are computed in prob_sup. The argument mod.output receives the object that stores the outcomes of the extr_outs function. save.df and interactive receive logical values, and determine if data frames with probabilities should be saved in the working directory (in .csv format) and if static plots should be converted into interactive plots using plotly (Sievert 2020), respectively.

prob_sup provides an overview of the selection candidates’ performance across environments, represented in a caterpillar plot containing the posterior genotypic main effects and their respective HPD intervals (Fig. 3a). The maximum a posteriori values are equivalent to marginal empirical BLUPs of Frequentist linear mixed models, assuming independent genotypic effects (see Appendix B). Then, it represents probabilities of superior performance and stability in lollipop plots as in Fig. 3b–d. For example, G36 was the candidate with the highest probability of superior performance (about 94%, Fig. 3b). In other words, there is only a 6% risk of poor performance, conditioned to the intensity of selection [$\text{Pr}({\hat{g}}_{j}\in \Omega |y)$]. The same interpretation is valid for the probability of superior stability: across locations (Fig. 3c), G23 has the greatest chance to perform equally (44%), while across regions (Fig. 3d), G15 has the most invariant performance (25%). Note how the results change across probability metrics. This illustrates the reason why plant breeders must have clear objective criteria before performing the analyses. If performance is preferred, Fig. 3b is the one to follow. Otherwise, if stability is the final goal, Fig. 3c and d must be prioritized.

**Fig. 4.**
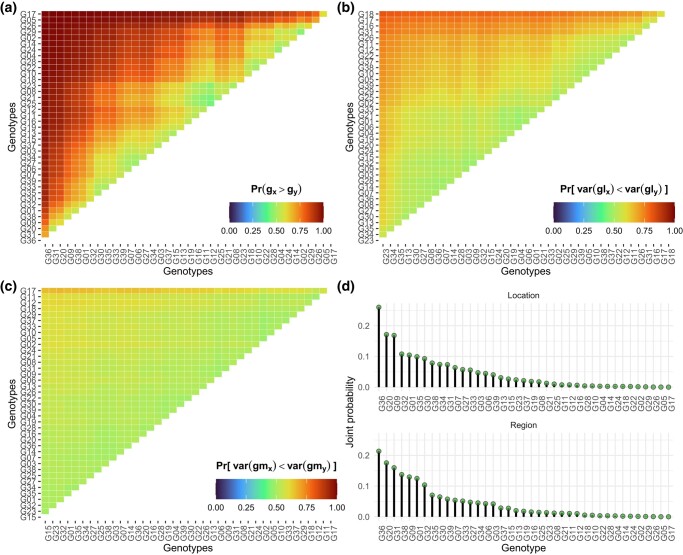
Pairwise probabilities of superior performance across locations a), superior stability across locations b), superior stability across regions c), and joint probability of superior performance and stability d). The heatmaps at a), b), and c) illustrate the probability of genotypes at the *x*-axis being superior to those on the *y*-axis. All plots were built with ggplot2 (Wickham 2016).

**Fig. 5.**
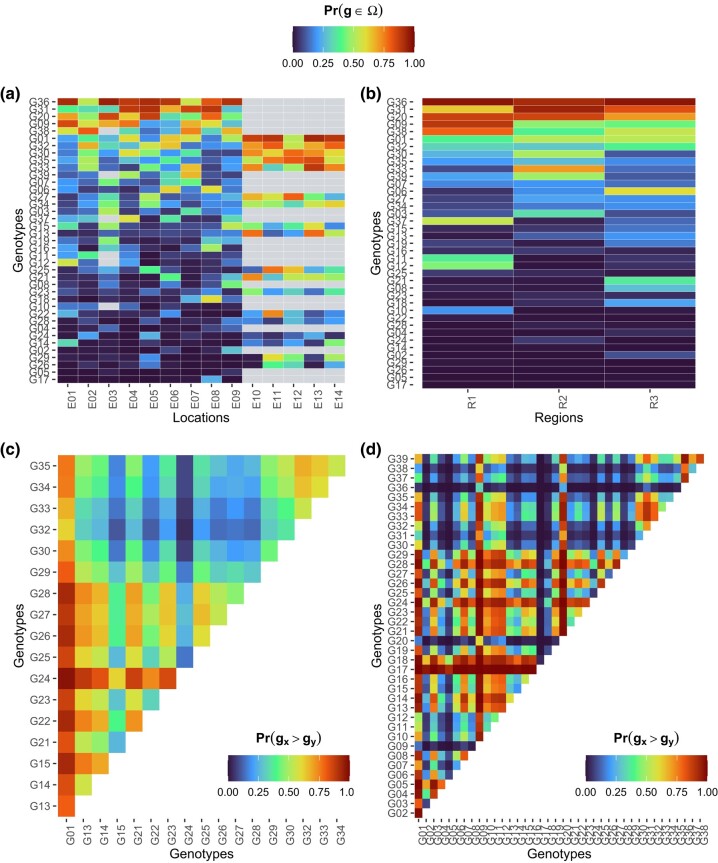
Heatmaps representing the specific probabilities of superior performance within locations a) and within regions b), and the pairwise probabilities of superior performance between genotypes evaluated in locations “E14” c), and in the region “R2” d). At a), the gray cells are locations where the genotype specified in the row was not evaluated. At c) and d), the probability of genotypes on the *x*-axis being superior to those on the *y*-axis are represented. All plots were built with ggplot2 (Wickham 2016).

In addition to the per se probabilities, we can compute pairwise probabilities for comparisons among genotypes (Fig. 4a–c). Suppose that G35 is a promising experimental genotype and that we want to investigate if it performs better than the commercial check G11. Across locations, G35 performs better than G11 at 80% of the times (Fig. 4a), and it has a more stable performance than G11 at 78% of the times (Fig. 4b). Then, there is evidence to hypothesize that genotype G35 is better than the commercial check. Finally, if breeders want to identify genotypes that simultaneously have high performance and stability, Fig. 4d is the one to analyze, as it contains the joint probability of superior performance and stability (circles). Note that the same genotype will hardly be the best in all probability metrics. Probabilities of superior performance and pairwise probabilities of superior performance are also available within locations and regions (Fig. 5), which is useful for specific recommendations.

### Concluding remarks

ProbBreed is a work in progress. The functionalities described in this paper can and will likely be improved, as well as other resources introduced in the future. Recommendations and suggestions from users are welcome. The computational time required to fit the Bayesian model is currently a limiting factor that should be emphasized. This time depends mainly on the processing capacity of the machine, the number of iterations, cores and chains set in bayes_met, and on the number of genotypes, locations, years, and regions (see Appendix B). For example, with 4 Markov chains running into 4 cores in parallel, the analysis of the Bayesian model fitted for the soy dataset took about 7.5 h to run with 40,000 iterations (3.6 h warming up and 3.9 h sampling).

In summary, ProbBreed is a user-friendly package for employing the risk/probability method proposed by Dias *et al*. (2022) for selecting genotypes in MET. We believe the package’s accessibility combined with the advantages of the Bayesian approach will encourage its adoption in the plant breeding community. The main advantage of using ProbBreed is effective decision-making for cultivar recommendation in MET. We recommend its usage mainly in late-stage breeding trials when a few dozen genotypes are evaluated in several environments.

## Figures and Tables

**Fig. A1.**
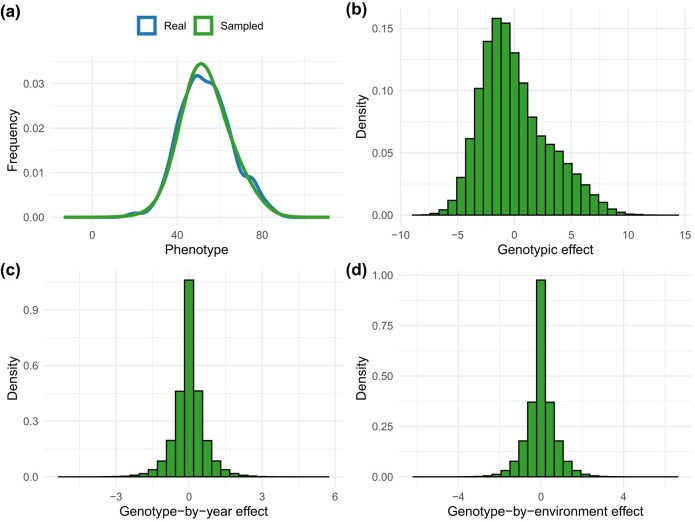
Density plot of the data generated in comparison to the distribution of the real data a), and histograms of the genotypic effect b), genotype-by-location effect c), and genotype-by-year effect d).

**Fig. A2.**
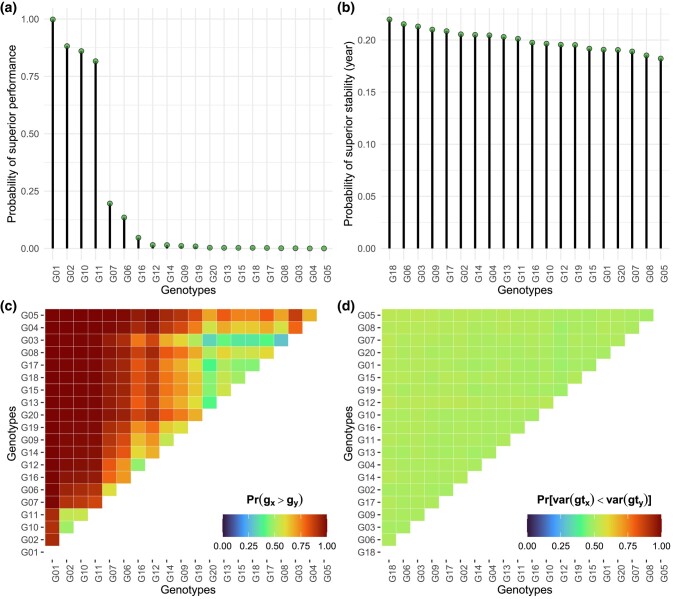
Probability of superior performance a) and stability considering the genotype-by-year interaction b), and pairwise probabilities of superior performance c) and stability d) considering the genotype-by-year interaction. At c) and d), the probability of genotypes on the *x*-axis being superior to those on the *y*-axis are represented.

**Fig. A3.**
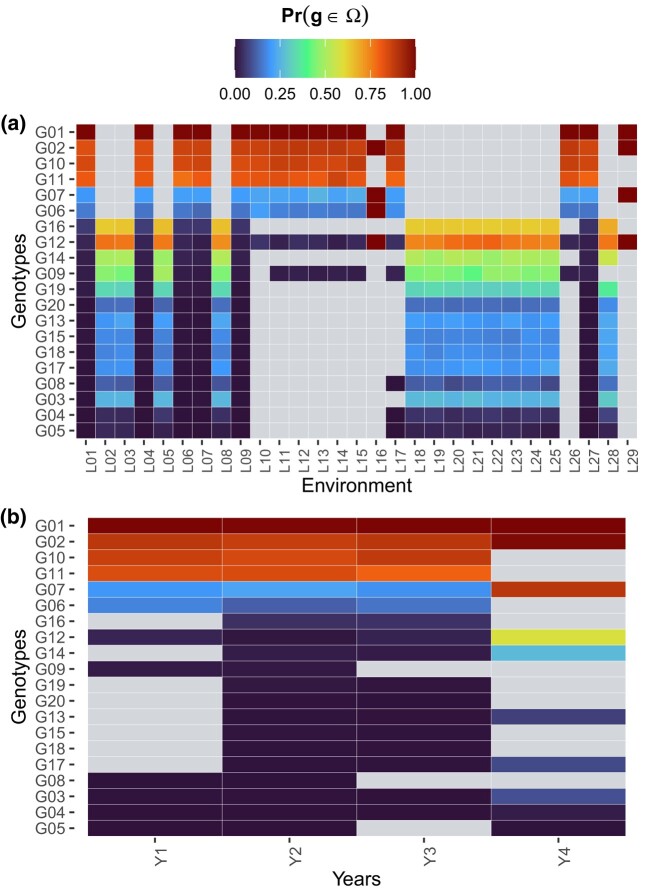
Probabilities of superior performance within locations a) and years b). The gray cells represent genotypes that were not evaluated in that specific location/year.

**Fig. A4.**
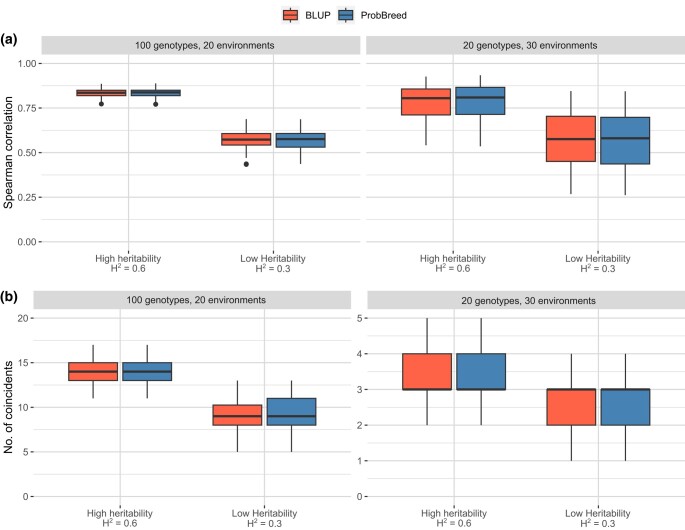
Comparisons between the simulated genotypic value and both the marginal probabilities of superior performance via ProbBreed and the BLUPs via ASReml-R: Spearman (rank) correlations a) and number of coincident selected genotypes b). The name of each facet and the text in the *x*-axes describe the simulated scenario. In b), we considered the top 20 and the top 5 genotypes in the scenarios with 100 genotypes assessed in 20 environments and 20 genotypes evaluated in 30 environments, respectively.

## Data Availability

The development version and the source code are available at https://github.com/saulo-chaves/ProbBreed. The package can be installed in R using the following commands (Box 4 for the development version, and Box 5 for the CRAN version): install.packages("devtools") devtools::install_github("saulo-chaves/ProbBreed") install.packages("ProbBreed") More details about the packages’ functionality are available at https://saulo-chaves.github.io/ProbBreed_site/.

## Appendix A: Multilocation–year model

The models described by Dias *et al*. (2022) consider information on locations or locations and breeding regions. As a novelty, we implemented in ProbBreed models that also consider the effect of years. To exemplify the usage of this model, we subsetted 4 years (2000 to 2003) of the USDA Northern Region Uniform Soybean Test (Krause *et al*. 2023). The dataset used as an example has the empirical best linear unbiased estimates (eBLUEs) of 20 genotypes evaluated at 29 locations. Six genotypes were evaluated in all 4 years, 4 were evaluated in 3 years, and the remainder were evaluated in only 2 years. In this situation, the conditional normal likelihood of the model is as follows:

(A1)
$$y_{\;jkh}∼N(E[y_{\;jkh}],\sigma )$$

with

(A2)
$$E[y_{\;jkh}]=\mu +g_{j}+l_{k}+t_{h}+gl_{\;jk}+gt_{\;jh}$$

where $y_{\;jkh}$ is the eBLUE of the jth genotype in the kth location and in the hth year, *μ* is the intercept, $g_{j}$ is the genotypic effect, $l_{k}$ is the effect of the location, $t_{h}$ is the effect of the year, $gl_{\;jk}$ is the genotype-by-location interaction effect, and $gt_{\;jh}$ is the genotype-by-year interaction effect. The prior and hyperprior probability distributions of this model follow the standard for all models in ProbBreed (see the description in *Results and discussion*). We ran the Bayesian model using 2000 iterations and 4 Markov chains. Leveraging the posterior distribution of this model, we computed the probabilities described in the Methods section considering a selection intensity of 20%.

The chains had an adequate mixing ($\hat{R}=1.002$), meaning that the generated data is a good representation of the empirical phenotypes (Fig. A1a). The histograms of the genotypic (Fig. A1b), genotype-by-year (Fig. A1c) and genotype-by-location effects (Fig. A1d) show the distribution of the posterior values of these effects.

Figure A2a shows that genotypes “G01”, “G02”, “G10”, and “G11” have a higher probability of performing across all locations. Supporting information is given by the pairwise probability of superior performance (Fig. A2c). The probability of superior stability between years has little variation among genotypes (Fig. A2d), meaning that they tend to have similar behavior in terms of stability across years. ProbBreed also provides other plots, such as the probability of superior stability and pairwise of superior stability across locations, and the joint probability of superior performance and stability, as described in the Results and discussion section.

Finally, we can investigate the performance of genotypes within locations and years (Fig. A3). Note that we are dealing with highly unbalanced data. In these situations, conclusions should be drawn with caution. Take the trials where the good performers “G01”, “G02”, “G10”, and “G11” were tested as examples (Fig. A2a). In trials with few genotypes (see “L10” to “L17”), depending on the selection intensity, “G01”, “G02”, “G10”, and “G11” will always appear among the selected candidates. Thus, we have to consider trials with more genotypes to determine if “G01”, “G02”, “G10”, and “G11” are, indeed, better performers than their peers. The same criteria should be applied when analyzing the performance of genotypes within each year (Fig. A2b).

### A.1. Computational requirements

Using a laptop with 10 threads, 8 GB of RAM, and a $12^{\mathrm{th}}$ Gen Intel Core i7-1255U processor with 1.70 GHz, it took 17 min to fit the Bayesian model. Ten of these minutes were dedicated to the warm-up iterations and the rest to the sampling iterations. We tracked the computational time to fit the Bayesian model using the get_elapsed_time function of rstan.

## Appendix B: Simulations

Stochastic simulations compared ProbBreed with traditional linear mixed models. Twenty datasets, for each scenario, were simulated according to the following hypothetical plans:

1. 100 genotypes evaluated in 20 environments, trait with high heritability ($H^{2}=0.6$).
2. 100 genotypes evaluated in 20 environments, trait with low heritability ($H^{2}=0.3$).
3. 20 genotypes evaluated in 30 environments, trait with high heritability ($H^{2}=0.6$).
4. 20 genotypes evaluated in 30 environments, trait with low heritability ($H^{2}=0.3$).

Scenarios 1 and 2 emulate the intermediate stages of a breeding program when several genotypes are tested in fewer environments, whereas scenarios 3 and 4 represent the final stage of a breeding program when there are fewer genotypes being tested in several environments. Phenotypes were simulated with the following model:

(A3)
$$y=Z_{1}e+Z_{2}b+Z_{3}g+\varepsilon$$

where y is the vector of simulated phenotypes, e is the vector of environmental effects, b is a vector of blocks within environments, g is the vector of genotypic effects and ε is the vector of errors. The capital letters represent the incidence matrices for their respective effects. e, b and ε were simulated to follow a normal distribution with mean zero and variance $\sigma_{e}^{2}$, $\sigma_{b}^{2}$ and $\sigma_{\varepsilon }^{2}$, respectively. We simulated g from a multivariate normal distribution with mean zero and variance-covariance $\left(\sigma_{g}^{2}J+\sigma_{ge}^{2}I\right)$, where $\sigma_{g}^{2}$ and $\sigma_{ge}^{2}$ are the variances of genotype and genotype-by-environment interaction effects, J is a matrix of ones, and I is an identity matrix. The dimensions of both matrices depend on the number of environments. Empirical estimates of variance components were obtained from Chaves *et al*. (2023) (Table A1).

**Table A1.** Estimates of the variance components (${\text{kg}}^{2}$) used for simulation.

Variance component	Estimate
$\sigma_{e}^{2}$	5.3137
$\sigma_{b}^{2}$	0.1075
$\sigma_{g}^{2}$	0.1215
$\sigma_{ge}^{2}$	0.3907
$\sigma_{\varepsilon }^{2}$	1.1420

$\sigma_{e}^{2}$ is the variance of the environmental effects, $\sigma_{b}^{2}$ is the variance of block effects, $\sigma_{g}^{2}$ is the genotypic variance, $\sigma_{ge}^{2}$ is the variance of the genotype-by-environment interaction effects, and $\sigma_{\varepsilon }^{2}$ is the residual variance.

In *ProbBreed*, we set Bayesian models with a default of 2000 iterations and 4 Markov chains. The simulated data was analyzed on a computer with 67 GB DDR4 of memory and a 12th Gen Intel Core i7-12100 processor with a base frequency of 2.10 GHz. We registered the average time to fit the models using the get_elapsed_time function of rstan. Once the models were fitted, we followed the pipeline described in the main text to obtain the probability metrics. Then, we performed the ranking correlation between estimated probabilities and simulated genotypic values. We also verified the number of coincident genotypes when selecting the top 20 genotypes via probabilities and simulated genotypic values for the datasets with 100 genotypes, whereas the top 5 genotypes were considered for the datasets with 20 genotypes. For comparison, the datasets were analyzed with a modified version of Model (A3) using a Frequentist linear mixed model with ASReml-R (version 4.2, The VSNi Team 2023), where e and b were considered as fixed effects. Using residual maximum likelihood, the Frequentist model predicted the best linear unbiased predictions (eBLUPs) of each genotype (Patterson and Thompson 1971; Henderson 1975). These eBLUPs were also compared with the simulated genotypic values using ranking correlations and detecting the coincidence among the selected genotypes, as previously described.

### B.1. Results

The results refer to the average across simulations. Both ProbBreed and BLUP yield more reliable results when analyzing traits with high heritability. The difference between rank correlations in the high- and low-heritability scenarios was 0.27 units and 0.22 units in datasets with 100 genotypes and 20 environments, and 20 genotypes and 30 environments, respectively (Fig. A4a). Similarly, the difference was evident in the number of coincident selected genotypes (Fig. A4b).

The results indicated no difference in selection/ranking between the probability of superior performance via ProbBreed and BLUP via the frequentist linear model regardless of the dataset size and the heritability level (Fig. A4). In fact, it is well known that when weakly informative priors are used, Bayesian and Frequentist models are equivalent (Sorensen and Gianola 2002). Thus, results from ProbBreed are comparable to the ones of other Frequentist methods in the context of multienvironmental trials. Despite the similarity, using probability metrics as criteria for selection has benefits that can aid in decision-making, such as the pairwise and joint probabilities of superior performance and stability. Future versions of the package may allow the consideration of different priors, which can increase the differences between ProbBreed and Frequentist models.

### B.2. Computational requirements

The models used to analyze the simulated datasets with 100 genotypes and 20 environments took about 10 h to fit, 6 dedicated to the warm-up iterations and 4 to the sampling iterations. Conversely, in the smaller simulated dataset needed, the computational time was reduced to 30 min (20 min warming up and 10 min sampling).
